# Mantle degassing along strike-slip faults in the Southeastern Korean Peninsula

**DOI:** 10.1038/s41598-019-51719-3

**Published:** 2019-10-25

**Authors:** Hyunwoo Lee, Heejun Kim, Takanori Kagoshima, Jin-Oh Park, Naoto Takahata, Yuji Sano

**Affiliations:** 1 School of Earth and Environmental Sciences, Seoul National University, 1 Gwanak-ro, Gwanak-gu, Seoul 08826 Republic of Korea; 2 Atmosphere and Ocean Research Institute, University of Tokyo, Kashiwa, Chiba 277-8564 Japan; 3 Institute of Surface-Earth System Science, Tianjin University, 92 Weijin Road, Nankai District, Tianjin 300072 P.R. China

**Keywords:** Geochemistry, Tectonics, Volcanology

## Abstract

On September 12, 2016, a M_L_ 5.8 earthquake hit Gyeongju in the southeastern part of the Korean Peninsula (SeKP), although the area is known to be far from the boundary of the active plate. A number of strike-slip faults are observed in heavily populated city areas (e.g., Busan, Ulsan, Pohang, and Gyeongju). However, dissolved gases related to the active faults have rarely been studied despite many groundwater wells and hot springs in the area. Here we report new results of gas compositions and isotope values of helium and carbon dioxide (CO_2_) in fault-related fluids in the region. Based on gas geochemistry, the majority of gas samples are abundant in CO_2_ (up to 99.91 vol.%). Measured ^3^He/^4^He ratios range from 0.07 to 5.66 Ra, showing that the mantle contribution is up to 71%. The range of carbon isotope compositions (δ^13^C) of CO_2_ is from −8.25 to −24.92‰, showing mantle-derived CO_2_ is observed coherently where high ^3^He/^4^He ratios appear. The weakening of faults seems to be related to enhanced pressures of fluids containing mantle-derived helium and CO_2_ despite the ductile lower crust underneath the region. Thus, we suggest that the SeKP strike-slip faults penetrate into the mantle through ductile shearing.

## Introduction

Helium and carbon dioxide (CO_2_) are favorable gas components to investigate sources of volatiles released at volcanic and hydrothermal regions. Helium and CO_2_ are closely related because CO_2_ is considered a carrier gas for helium transport^1^. Isotopic compositions of both helium and CO_2_ provide information about mantle-derived volatiles. First, helium has ^3^He (primordial) and ^4^He (radiogenic) and gives reference values for geochemical reservoirs, such as mid-ocean ridge basalts (MORB, 8 ± 1Ra) and crust (0.02Ra), where the ^3^He/^4^He ratio of air (1Ra) is 1.389 × 10^−6^ (ref.^2^). For CO_2_, it is reported that the range of carbon isotope compositions (δ^13^C versus the standard of Vienna Pee Dee Belemnite (V-PDB)) of MORB, carbonate, and organic sediment are between 0 and −30‰^3^.

On the basis of helium and CO_2_ geochemistry, many studies have reported that continental rift fault zones (e.g., Eger Rift and East African Rift) release mantle-derived volatiles through fault-related springs^4,5^. Additionally, diffusive soil degassing of mantle-derived CO_2_ has been observed in nonvolcanic young extensional fault zones^6^. Strike-slip faults are also regarded permeable pathways for degassing of mantle-derived volatiles (e.g., San Andreas and North Anatolian faults). However, magmatism is believed to be absent^7–11^.

Fluids are thought to enhance pore fluid pressures in association with fault weakening^7,12^. In the San Andreas fault zones, ref.^7^ proposed that mantle fluids are related to fault weakening. Subsequently, ref.^8^ found that shear zones with high strain rates discharge more mantle fluids through the ductile lower crust. Also, seismicity likely coincides with mantle-derived fluids according to many studies. Deep earthquakes (<35 km depths) were observed in East Africa, together with mantle-derived volatiles released at normal faults^5,6^. Strike-slip faults show a correlation between deep earthquakes and high ^3^He/^4^He ratios, reported in the Newport-Inglewood fault zone, Southern California^13^.

**Figure 1:**
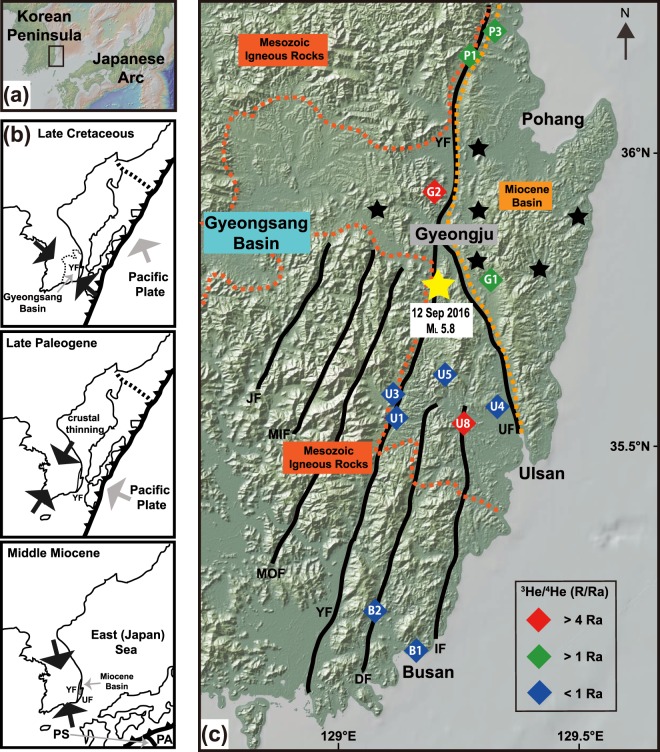
Locations of sampling and strike-slip faults in the Southeastern Korean Peninsula (SeKP). (**a**) Location of the study area. The map showing the Korean peninsula and the Japanese arc was created using GeoMapApp 3.6.10 (ref.^56^). (**b**) Tectonic evolution of the Korean peninsula between Late Cretaceous and Middle Miocene (modified based on ref.^17^). The movement of the plates and the direction of the stress are shown on the basis of ref.^17^. Abbreviated names of the subducting plates and faults are displayed. PA: Pacific plate; PS: Philippine Sea plate; YF: Yangsan fault; UF: Ulsan fault. (**c**) Helium isotope compositions of sampled sites. The Gyeongsang is displayed with the Mesozoic igneous rocks (dark orange dashed lines) and the Miocene basin (bright orange dashed lines)^17^. Different colors of the sampling sites represent that samples have different contributions of mantle-derived volatiles. Sampling IDs are labeled on the symbols. Red diamonds have more than 50% of the mantle-derived helium (8 Ra). Green and blue colored samples contain less amounts of ^3^He. The yellow star is the location of the M_L_ 5.8 Earthquake, which occurred in Gyeongju on September 12, 2016. The black stars show earthquakes (M > 3) reported by the Korea Meteorological Agency near the Gyeongju area since 1986. Major cities (Busan, Ulsan, Pohang, and Gyeongju) are displayed on the map. Abbreviated names of the faults are shown. YF: Yangsan fault; UF: Ulsan fault; IF: Ilkwang fault; DF: Dongrae fault; MOF: Moryang fault; MIF: Milyang fault; JF: Jain fault.

The southeastern Korean Peninsula (SeKP) is above the Gyeongsang Basin which was formed in the Cretaceous period. The Gyeongsang Basin is an area related to the Cretaceous intrusive and Tertiary volcanic activities^14^. During the early Cretaceous period, the Gyeongsang Basin formed a number of pull-apart basins and strike-slip faults in association with the subduction of the Izanagi plate, which resulted in the accumulation of lacustrine siliciclastic sediments^15,16^. In the late Cretaceous, the subduction of the Pacific plate caused the NW-SE trending compressive stress in the Korean peninsula^17^ (Fig. 1b). There were dike swarms in the southeastern part of the Gyeongsang basin in the early Paleogene^18,19^. It is known that the Pacific plate subduction was further directed to the west, resulting in the NE-SE compression and crustal thinning of the East (Japan) sea during the late Paleogene^17^. The opening of the East (Japan) sea began at ~25 Ma with normal faulting and dike swarms and formed the Miocene basins in the SeKP area^20^. Due to the subduction of the Philippine Sea plate, the opening was ceased at ~16 Ma (Fig. 1b), and compression appeared in the faults and basins^17^. According to the Quaternary fault slip data, the SeKP faults in are under compressive stress and are also suggested to be due to the subduction of the plates of the Pacific and Philippine seas and the collision of the Eurasian continent of India^14,17^.

In the SeKP region, there are a number of NNE-SSW trending dextral strike-slip faults, such as Yangsan, Ulsan, Ilkwang, Dongrae, Moryang, Milyang, and Jain faults (Fig. 1c). The faults are believed to be active faults due to recent earthquakes occurring in this region, such as the M_L_ 5.8 Gyeongju Earthquake at 11:32 a.m. on September 12, 2016 after the M_L_ 5.1 foreshock at 10:44 a.m. on the same day (Fig. 1c). It is known that high geothermal heat flow is present in this area, and hot springs and groundwater circulation associated with the fault are shown. However, dissolved gases in fluids related to the active fault lines have rarely been studied. Moreover, geochemistry and isotopic compositions of dissolved gases in fluids are poorly known to better understand the faulting mechanism and seismic activities in the SeKP region. Here we report new results of gas geochemistry, ^3^He/^4^He ratios and δ^13^C-CO_2_ of dissolved gases in fault-related fluids in the SeKP area to constrain sources of volatiles in association with fault weakening and seismic activities.

## Results and Discussion

### Gas geochemistry of the fault fluids

We report the first results of gas geochemistry in fluids released along the SeKP fault zones (Tables 1 and 2). The SeKP fault gases are dominated by the atmospheric sources (e.g., air and ASW) as shown on the N2-Ar-He ternary diagram that simply distinguishes sources of volcanic and geothermal gases^21^. Measured He and Ar contents (0.4 to 614 ppm and 0.02 to 1.42 vol.%, respectively) produce lower He/Ar ratios (0.0004 to 0.05) than that of MORB (He/Ar = 2, ref.^22^), plotting data points towards the air/ASW side on the ternary diagram. This is commonly observed in spring gas compositions in nonvolcanic regions such as forearc areas (e.g., refs.^23,24^).

**Table 1:** Sampling information and gas compositions of the SeKP fluids.

Sample ID	Latitude (°N)	Longitude (°E)	Sampling Date	Type^a^	T (°C)	pH	well depth (m)
G1	35.784863	129.32511	2018-01-19	GW	9.8	6.4	150
G2	35.92999	129.20593	2019-02-11	spring	12.5	6.4	—
U1	35.552748	129.12584	2018-01-18	GW	13.7	7.5	200
U3	35.59307	129.11579	2018-01-18	GW	15.7	7.8	296
U4	35.570406	129.33711	2018-01-18	GW	13.4	6.0	130
U5a	35.623588	129.22283	2018-01-18	HS	36.0	10.3	750
U5b			2018-11-11	HS	30.0	9.3	750
U8a	35.537891	129.25591	2018-11-11	HS	24.1	6.3	650
U8b			2019-02-19	HS	23.9	6.3	
P1a	36.159214	129.27773	2018-01-19	HS	51.8	9.2	700
P1b			2018-11-12	HS	53.3	9.4	700
P3	36.194684	129.32737	2018-01-19	GW	16.1	6.5	180
B1a	35.161686	129.16648	2018-01-17	HS	54.6	7.0	153
B1b			2018-11-13	HS	56.7	6.7	153
B2a	35.22062	129.08095	2018-11-13	HS	63.7	7.4	230
B2b			2018-01-17	HS	60.1	7.8	230

^a^GW: groundwater well; HS: hot spring.

**Table 2:** Gas and isotopic compositions of the SeKP fluids.

Sample ID	He	CH_4_	N_2_	O_2_	Ar	CO_2_	He/Ar	N_2_/Ar	N_2_/He	^4^He/^20^Ne	^3^He/^4^He			δ^13^C-CO_2_^a^	error
	ppmv	(vol.%)									R/Ra	error	Rc/Ra		
G1	4	0.02	77.29	21.55	0.99	0.15	0.0004	78	196,022	0.4	1.09	0.02	1.20	−24.92	0.29
G2	80	0.01	10.59	0.03	0.21	89.16	0.04	50	1,316	38	5.51	0.01	5.54	−12.18	0.30
U1	126	—	6.62	—	0.26	93.10	0.05	25	524	0.4	0.84	0.02	0.55	−14.50	0.29
U3	175	0.04	23.47	3.31	0.60	72.56	0.03	39	1,344	0.7	0.70	0.02	0.55	−19.80	0.27
U4	362	0.08	45.65	5.51	1.26	47.46	0.03	36	1,263	0.3	0.96	0.03	0.87	—	—
U5a	348	0.06	75.51	11.89	1.22	11.29	0.03	62	2,168	12	0.07	0.03	0.05	−23.73	0.32
U5b	4	0.04	78.30	20.61	0.93	0.12	0.0005	85	175,614	2.8	0.17	0.04	0.09	−23.84	0.35
U8a	4	0.01	0.66	—	0.02	99.31	0.02	29	1,742	17	4.84	0.10	4.90	−8.53	0.10
U8b	0.4	—	0.08	0.00	0.00	99.91	0.02	36	2,067	38	5.66	0.01	5.69	−8.25	0.30
P1a	61	—	82.58	16.46	0.95	—	0.01	87	13,445	19	1.38	0.03	1.39	—	—
P1b	614	0.15	95.31	—	1.19	3.25	0.05	80	1,553	33	1.27	0.04	1.27	−20.24	2.69
P3	263	—	63.76	—	1.15	35.07	0.02	56	2,423	0.5	1.34	0.03	1.68	—	—
B1a	64	0.04	79.59	17.83	0.98	1.57	0.01	81	12,510	24	0.34	0.01	0.33	—	—
B1b	87	0.09	83.43	12.46	0.92	3.07	0.01	91	9,560	39	0.31	0.04	0.30	−22.67	0.57
B2a	496	0.07	95.80	0.00	1.42	2.66	0.04	68	1,930	37	0.34	0.03	0.34	−20.80	0.55
B2b	12	0.02	78.49	20.28	0.88	0.33	0.001	89	65,358	25	0.46	0.01	0.46	−16.69	0.27

^a^δ^13^C-CO_2_ values of U4, P1, P3, and B1a were not measured due to small amount of gas in the samples.

In the majority of the SeKP fault fluid samples, dissolved CO_2_ concentrations are higher than in the atmosphere (up to 99.91 vol.%, Table 2). CO_2_ is the most abundant component for G2, U1, U3, U4, U8 and P3, ranging from 35.07 to 99.91 vol.%. The other samples (G1, U5, P1, B1 and B2) have abundant N_2_ (>75 vol.%) and O_2_ (11.89 to 21.55 vol.%), except for P1b (N_2_ = 95.31 vol.%) and B2b (N_2_ = 95.80 vol.%). The samples displaying both abundant N_2_ and O_2_ are likely to be contaminated by air during sample procedures at the sites. Thus, the SeKP faults are possibly permeable for CO_2_ transport as shown in other non-volcanic faulted areas, such as the San Andreas fault^25,26^, the North Anatolian fault^9^, and the East African Rift^5,6^.

**Figure 2:**
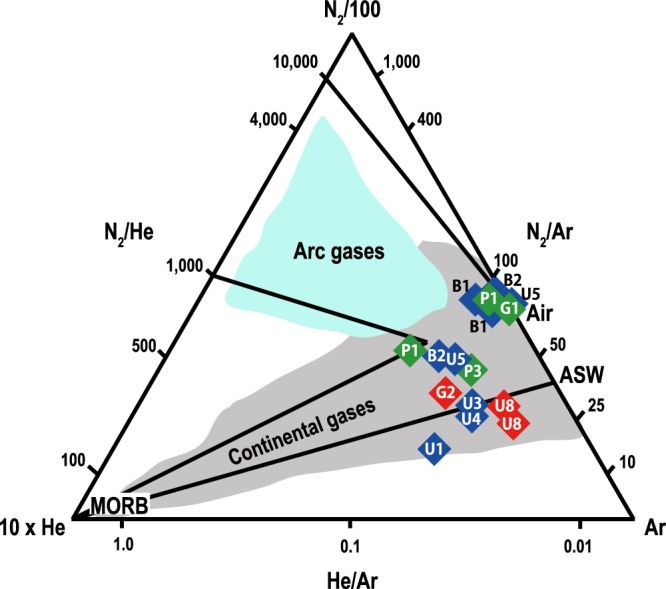
N_2_-Ar-He ternary plot. Relative N_2_, Ar, and He abundances of dissolved gases in fault-related fluids in the SeKP region are displayed. Referenced data of continental gases^5^ and arc gases^21^ are shown. The SeKP gases are plotted on the trend for continental gases, mixed by MORB and atmospheric components (air and ASW).

The SeKP fault gases follow the trend of continental gases rather than the subducting slab components (Fig. 2). The N_2_-Ar-He relative abundances show a mixing trend between MORB and atmospheric sources. The CO_2_-rich samples generally have higher He/Ar ratios from 0.02 to 0.05 than the values of the N_2_-rich samples (He/Ar = 0.0004 to 0.04). Moreover, they have lower N_2_/Ar ratios ranging from 25 to 56, which are close to the ASW composition (38 at 20 °C, ref.^27^). N_2_/Ar ratios of the N_2_-rich gases range from 62 to 91, which is close to that of air (84, ref.^27^) and plot towards air on the N_2_-Ar-He ternary diagram (Fig. 2). N_2_/He ratios of all samples (524 to 196,022) are higher than the MORB ratio (N_2_/He = 150, refs.^22,28^). The samples (G1, U5b, and B2a) with higher O_2_ contents (> 20 vol.%) have higher N_2_/He ratios (65,358 to 196,022) like air (N_2_/He = 148,900, ref.^2^). N_2_/He ratios (524 to 13,445) of the other samples are still lower than that of the subducting slab (N_2_/He = 20,000, ref.^29^).

### Mantle-derived helium degassing through the SeKP fault zones

**Figure 3:**
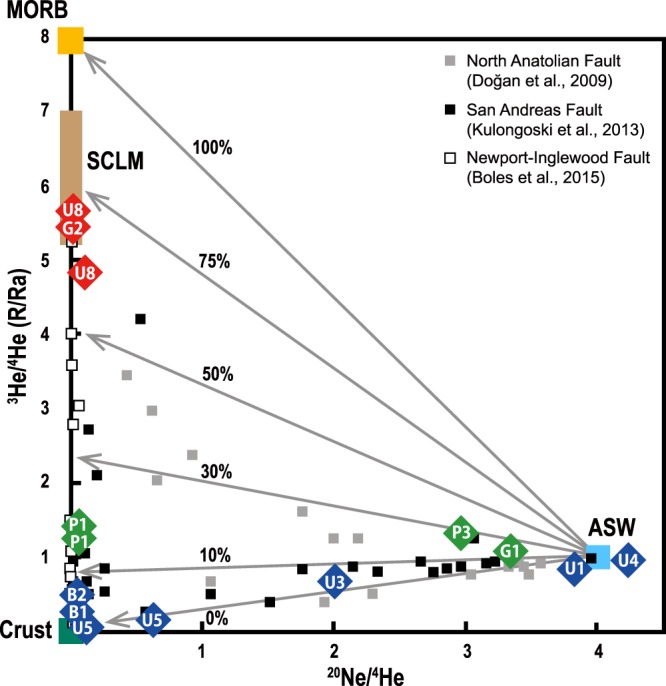
^3^He/^4^He (R/Ra) versus ^20^Ne/^4^He plot. Three reference values of MORB, crust and ASW are displayed as well as subcontinental lithospheric mantle (SCLM, 6.1 ± 0.9 Ra, ref.^31^). Each gray line shows linear mixing trends between mantle/crust and ASW with different MORB (8 Ra) contribution ratios. Referenced data of other strike-slip fault zones in the world are shown.

Based on the range of measured ^3^He/^4^He ratios (0.07 to 5.66 Ra) and inverse values of measured ^4^He/^20^Ne (0.34 to 38.5) in the SeKP fault gases, there are linear relationships between helium sources and the atmospheric component (ASW). All of the groundwater samples have lower ^4^He/^20^Ne ratios from 0.3 to 0.7 due to their shallower well depths (130 to 296 m), enhancing air contribution. Nevertheless, the other samples with elevated ^4^He/^20^Ne ratios (2.8 to 38.5) show that the ^3^He/^4^He ratios of those samples largely well maintain the sources (Fig. 3). It is attributed that ^4^He flux can be derived from both mantle and crust^5,30^.

We divided the SeKP fault fluids into three groups based on mantle-derived helium portions (Figs. 1c and 3). The first group shows that ^3^He/^4^He ratios are > 50% of MORB-derived helium. They were found at G2 (^3^He/^4^He = 5.51 Ra) near the Yangsan fault zone and U8 (^3^He/^4^He = 4.84 and 5.66 Ra) near the Ilkwang fault zone with 61% to 71% of MORB-derived helium (Figs. 1c and 3). Also, it is probable that the values are rather close to the subcontinental lithospheric mantle (SCLM) ratio which is 6.1 ± 0.9 Ra (ref.^31^), but lower than the average of arc volcano, 7.4 ± 1.3 Ra (ref.^32^). These ^3^He/^4^He ratios are comparable to reported values of other strike-slip fault systems, such as the San Andreas (^3^He/^4^He max = 5.3 Ra, ref.^11^), North Anatolian (^3^He/^4^He max = 4.6 Ra, refs.^9,33^), and Karakoram (^3^He/^4^He max = 2.2 Ra, ref.^34^) fault zones (Fig. 3).

The second group has MORB-derived helium portions between 10 and 50%, implying that the mantle-derived helium has interacted with the crust during upward migration. This type is found at P1a and P1b (16% and 17% of the MORB-derived helium with elevated ^4^He/^20^Ne ratio of 19 and 33) which is located at the Pohang section of the Yangsan fault (Fig. 1c). However, G1 and P3 are atmospheric (^4^He/^20^Ne = 0.43 and 0.49) likely due to shallower well depths (150 m and 180 m, respectively) than P1 of 700 m (Fig. 3), although they have ^3^He/^4^He ratios of 1.09 and 1.34 Ra, respectively.

The last group seems to be contributed somewhat by radiogenic helium from the crust (Figs. 1c and 3), showing < 10% of MORB-derived helium. This group has ^3^He/^4^He ratios from 0.07 to 0.96 Ra with Rc/Ra values from 0.05 to 0.87 (Table 2). It is known that the SeKP strike-slip faults are developed by cutting the Cretaceous granitic rocks (~75 Ma, ref.^35^, Fig. 1c) in association with abundant radiogenic ^4^He production by the alpha decay of U and Th. It is experimentally reported that granitic rocks release radiogenic ^4^He during deformation^36,37^. However, excluding groundwater samples with low ^4^He/^20^Ne ratios possibly due to air contamination, the hot spring samples in this group have ^3^He/^4^He ratios > 0.1 Ra, except for U5a (0.07 Ra), indicating that the majority of these samples still have mantle-derived helium according to ref.^1^.

### CO_2_ sources of the SeKP fault fluids

**Figure 4:**
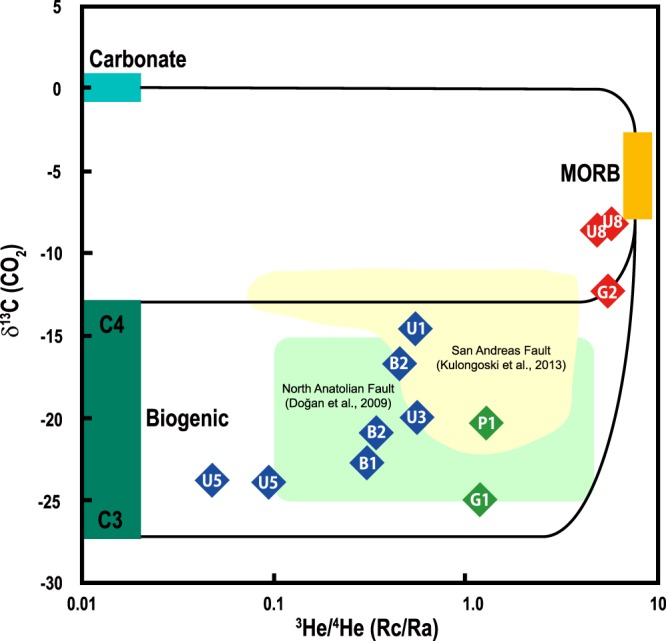
δ^13^C-CO_2_ and corrected ^3^He/^4^He (Rc/Ra) plot. Mixing lines are defined between the MORB and crustal (carbonate and biogenic carbon) end-members. δ^13^C values of MORB and carbonate components are from ref.^3^. The biogenic source is displayed based on the mean δ^13^C values of C3 and C4 plants^38^. Referenced data of the San Andreas and North Anatolian fault zones is displayed.

Heavier δ^13^C values (−8.25‰ to −12.18‰) of CO_2_ are observed where higher ^3^He/^4^He ratios are found (Fig. 4). The possible scenario is that the δ^13^C values of G2, U8a and U8b with higher ^3^He/^4^He ratio (Rc/Ra = 4.90 to 5.69) are closer to the MORB value (δ^13^C = −6.5 ± 2‰, ref.^3^) due to less contribution of biogenic CO_2_ than other samples (δ^13^C = −14.50 to −24.92‰; Rc/Ra = 0.05 to 1.68) (Fig. 4). It is attributed that mantle-derived CO_2_ plays a role as a carrier gas for ^3^He (ref.^1^). In a number of tectonically active areas, fault-related springs discharge mantle-derived CO_2_ together with ^3^He, such as the East African Rift, Eger Rift and San Andreas Fault^4–6,10,11^. δ^13^C values of other samples fall between the mean values of C3 (δ^13^C = −27‰) and C4 (δ^13^C = −13‰) plants^38^, which is similar to the results of other strike-slip faults, such as the San Andreas and North Anatolian fault zones releasing biogenic soil CO_2_ with the range of δ^13^C from −11.87 to −24.0‰ (refs.^9,10,25,26^) (Fig. 4). The SeKP fault zones are developed in the lacustrine sedimentary Gyeongsang Basin formed in the Cretaceous period^14^, containing organic materials with δ^13^C values of −20.7 to −26.4‰ **(**ref.^39^**)**. Miocene basins formed by the Yangsan and Ulsan faults^14^ may have C4 plants-derived sediments, which have heavier biogenic δ^13^C values than the C3 plant likely due to global vegetation change in the Miocene and Pliocene periods^40^. However, The interaction of CO_2_ and water could result in carbon isotope fractionation between gaseous CO_2_ and dissolved HCO_3_^−^ and/or CO_3_^2−^, depending on the pH of water samples^41^. The pH range of the SekP fluids are between 6.0 and 10.3 (Table 1), implying some portion of CO_2_ was not sufficiently degassed when dissolved gases were extracted from fluid samples. In the Gyeongju area, δ^13^C values of dissolved inorganic carbon (DIC) in groundwater samples have been reported with the range of −12.72 to −17.17‰ (ref.^42^), which is included in the range of our results (Fig. 4). Thus, the carbon isotope fractionation could be insignificant based on the differences in δ^13^C values between CO_2_ and DIC.

### Fault weakening and strike-slip shearing into the lithospheric mantle

The fault zones in the SeKP region may be weakened in association with fluids containing mantle-derived volatiles. Ref.^43^ observed fault gouges containing clay minerals to reduce friction of the Yangsan fault, providing evidence for fault weakening. It is well known that highly pressured fluids interacting with country rocks can invoke fault weakening^12,44^. Ref.^7^ also proposed that the San Andreas Fault weakening can be related to ^3^He-rich mantle fluids which are incorporated into the seismogenic zone to enhance pore fluid pressures. On the basis of mantle-derived helium (^3^He/^4^He ratio up to 5.3Ra) and CO_2_ (δ^13^C = 0 to −10‰), ref.^11^ proposed mantle degassing in the Newport-Inglewood strike-slip fault zone, where magmatism is absent. In the case of the Gyeongju area (Fig. 1c), ref.^45^ observed a low velocity layer in the seismogenic zone (<15 km, ref.^46^), with depths of 4 to 14 km. Ref.^47^ suggested that Cretaceous adakite intrusions and/or the high geothermal gradient are possible causes for the low velocity layer. However, the intrusions can be sources to produce radiogenic ^4^He as discussed above, indicating the hypothesis is implausible due to the elevated ^3^He/^4^He ratio (5.51 Ra for G2) in the region. Even though the SeKP region shows high heat flow values due to shallow Moho depths (~80 mW/m^2^, ref.^47^), the low velocity zone only exists in a limited depth range (4 to 14 km). Instead, we propose that the low velocity zone may contain aqueous fluids to weaken the fault zones. Ref.^48^ showed a low velocity zone within the crust beneath northeast Japan (>10 km depths), suggesting the presence of aqueous fluids related to the 2008 Iwate earthquake (M7.2). Therefore, mantle-derived volatiles observed near the localities of the M_L_ 5.8 Gyeongju earthquake (Fig. 1c) provide information about fluids related to mantle degassing for the seismically active fault.

Mantle degassing in the SeKP region illustrates that the fault zones appear to penetrate into the lithospheric mantle. The Moho depths of the SeKP region ranges between 26 and 32 km based on S-wave velocities^45^, with the seismogenic depth of 15 km (ref ^46^). Hence, the brittle-ductile transitions zone may be located at the depth of 15 km, which is consistent with the lower crustal thickness approximately ranging from 15 and 30 km (ref.^45^). The ductile lower crust is thought to be impermeable for mantle-derived helium transport^7,8,44^. To resolve the mantle-derived helium migration through the impermeable lower crust, active degassing via magmatism^49^ and diffusive transport at extensional settings^30^ have been suggested. However, magmatism underneath the SeKP region is unlikely because volcanism has been ceased in the Miocene period. Also, diffusion of mantle-derived helium is ambiguous for strike-slip faults despite magmatic helium and CO_2_ found in the continental rift fault zones^4–6^. The alternative scenario for penetration of mantle-derived volatiles is to have permeable pathways, such as deep faults through the lower crust^7,11,13^. However, deep earthquakes (depths > 15 km) are poorly observed in the SeKP region, although GPS data reported by ref.^50^ shows higher shear strain rates occurring in the area. Therefore, we propose high strain localized shearing through the lower crust and lithospheric mantle. Ref.^8^ suggested that the San Andreas fault zones release mantle fluids at high strain shear zones through the ductile lower crust based on the correlation between ^3^He/^4^He ratios and GPS strain rates. Moreover, ref.^51^ showed that highly deformed ultramylonite peridotites are enriched in mantle helium. Furthermore, higher helium contents were found in the highly sheared peridotites^52^.

## Conclusions

We first report the results of gas geochemistry, ^3^He/^4^He ratios and δ^13^C of CO_2_ from fluids in the SeKP fault system (Fig. 1c). The N_2_-Ar-He abundances illustrate that the SeKP fault gases are unlikely to be the arc gases but are likely derived from the mantle source like other continental gases despite some samples are almost atmospheric owing to shallow well depths (Fig. 2). Mantle-derived helium (^3^He/^4^He > 0.1 Ra) are observed in entire the SeKP fault zones although heavier δ^13^C values appear only where higher ^3^He/^4^He ratios are shown. These features are also observed in other strike-slip fault zones (e.g., North Anatolian, San Andreas, and Newport-Inglewood fault zones) in the world (Figs. 3 and 4). The seismically active SeKP faults are weakened, which can be attributed to high pressures of fluids containing mantle-derived volatiles like the San Andreas fault area. In the SeKP region, there should be potential pathways for mantle-derived volatiles to migrate through the ductile lower crust because the Moho depth (~30 km) is deeper than the possible brittle-ductile transitions zone (~15 km) beneath this area. Thus, we suggest that the SeKP strike-slip faults extend in the form of ductile shear zones to the lithospheric mantle below the seismogenic depth (~15 km).

## Sampling and Analytical Methods

Samples were collected in the Gyeongju, Ulsan, Pohang, and Busan areas (Table 1 and Fig. 1). Fluid samples containing dissolved gases were delivered into glass containers for measuring gas compositions and carbon isotopes of CO_2_. Copper tubes were also used to sample fluids for noble gas analyses in order to prevent helium-loss through silicate glasses. Dissolved gases in fluids were extracted by using a high vacuum pumping system, and all analyses were carried out in the Atmosphere and Ocean Research Institute (AORI), the University of Tokyo. Concentrations of CO_2_, N_2_, O_2_, CH_4_, Ar, and He in dissolved gases were obtained by a quadrupole mass spectrometer (QMS) using a secondary electron multiplier (Pfeiffer Prisma QMS 200). The analytical errors of relatively abundant gas components (CO_2_, N_2_ and O_2_) and minor gases (CH_4_, Ar and He) were about 10% and 30% at 2σ, respectively, which were determined by repeated measurements of air. Helium isotope ratios (^3^He/^4^He) were measured by a VG-5400 noble gas mass spectrometer. He and Ne were purified by titanium getters (at 400 °C) and charcoal traps at liquid nitrogen temperature. Neon was trapped by a cryogenic trap (at 40 K) after measuring ^4^He/^20^Ne ratios by an on-line QMS. Calibration of He isotope ratios was conducted by using the internal He standard of Japan^53^. Measured helium isotope compositions were corrected for atmospheric helium by using measured ^4^He/^20^Ne ratios since ^20^Ne is assumed to be mostly atmospheric^54^. As described in ref.^55^:

1 $${\rm{Rc}}/{\rm{Ra}}=[{{(}^{3}{\rm{He}}{/}^{4}{\rm{He}})}_{{\rm{measured}}}-{\rm{r}}]/(1-{\rm{r}})$$

2 $${\rm{r}}={{(}^{4}{\rm{He}}{/}^{{\rm{20}}}{\rm{Ne}})}_{{\rm{ASW}}}/{{(}^{{\rm{4}}}{\rm{He}}{/}^{{\rm{20}}}{\rm{Ne}})}_{{\rm{measured}}}$$

where Rc/Ra is the corrected ^3^He/^4^He ratio, and (^4^He/^20^Ne)_ASW_ is the ^4^He/^20^Ne ratio of air saturated water (ASW). Analytical errors for ^3^He/^4^He and ^4^He/^20^Ne ratios are about 3.5% and 5% (1σ), respectively. Carbon isotope compositions (δ^13^C) of CO_2_ which is extracted from fluids were measured by an isotope ratio mass spectrometer (IsoPrime 100) with an elemental analyzer. The analytical error was approximately 0.3‰ (2σ).
